# Medical and Physical Intelligence

**Published:** 1804-09-01

**Authors:** 


### MEDICAL AND PHYSICAL INTELLIGENCE.

[ FOREIGN AND DOMESTIC. ]

Mr. SCHAUB, of Cassel, has made some observations on the use of charcoal, in which the experiments of Mr. Lowitz with this substance are for the most part confirmed. Mr. S. employed charcoal with advantage for clarifying different liquors, and it also served to whiten tartarous acid and different solutions of salts. Meat, which had acquired the highest degree of putrefaction, on being rubbed with charcoal powder, lost entirely the putrid stench, and only disengaged the smell of ammonia. Mr. Schaub has preserved venison for above six weeks in an unaltered state, by covering it with charcoal powder. On adding to honey, dissolved in water, a certain quantity of charcoal powder, it will lose its colour and smell; brandy is likewise deprived of its particular smell by shaking it with charcoal powder, and afterwards distilling it. The residuum of sulphuric ether, on being mixed with charcoal, becomes clear and transparent like water. The odours of several substances, as valerian, galbanum, balsamus Peruvianus, musk, &c. have been diminished, or have entirely ceased by the addition of charcoal powder. Crude tartar, on being boiled with charcoal, yields pure and white crystals.

---

Dr. HUNOLD, in his observations on the use of charcoal in different diseases, has found it of great effect in herpetic exanthemata, particularly those which arise from the frequent eating of salt meat. For this purpose he mixed charcoal powder with as much rum as rendered it the consistency of a soft plaster, which he applied to the diseased spots, whereby the eruptions gradually dried, and then disappeared. Charcoal powder mixed with an equal equal quantity of pulvis cortic. Peruviani, is successfully employed for scorbutic gums, the bleeding of which will certainly cease, and the disagreeable smell of the breath be greatly diminished, by gently rubbing the gums with this powder. He likewise administered the charcoal powder with much success in putrid fevers, in a dose of half a drachm, to be taken six times a day. In foul ulcers of the legs it proved likewise very serviceable, and he found it also to be a useful antiseptic applied on gangrenous parts.

---

St. Thomas's and Guy's Hospitals.

The Lectures for the ensuing season, at these adjoining Hospitals, will commence on the 1st of October.

St. Thomas's Hospital.—Anatomy, human and comparative, by Mr. Cline and Mr. Cooper; Principles and Practice of Surgery, by Mr. Cooper.

Guy's Hospital.—Practice of Medicine, by Dr. Babington and Dr. Curry.

Chemistry and Experimental Philosophy, by Dr. Babington and Mr. Allen.

Theory of Medicine and Materia Medica, by Dr. Curry.

Midwifery and the Diseases of Women and Children, by Dr. Haighton.

Physiology, or Laws of the Animal Economy, by Dr. Haighton.

Occasional Clinical Lectures on select Medical Cases, by Drs. Babington, Curry, and Marcet.

Some time in the winter will be given a Course of Lectures on the Structure and Diseases of the Teeth, by Mr. Fox, surgeon-dentist; and on the Veterinary Art, by Mr. Coleman, Professor at the Veterinary College.

These several Lectures are so arranged, that no two of them interfere with each other in the hours of attendance; and the whole is calculated to form a complete course of medical and chirurgical instruction. Terms, and other particulars, to be learnt from Mr. Stocker, apothecary to Guy's Hospital, who is also empowered to enter gentlemen as pupils, to such of the lectures as are delivered at Guy's. Among the numerous improvements that are now carrying on at Guy's Hospital, intended to facilitate and to increase the acquisition of medical and chirurgical science to pupils, not only a new, enlarged, and commodious Lecturing Theatre has been built, so contrived as to admit of the several chemical processes being carried on before the class; but there is already in considerable forwardness an extensive Library and Museum, in which, along with the valuable collection of books belonging to the Physical Society, there will be deposited the various morbid preparations that are daily accumulating, with a correct account of the respective cases to which they belonged, so as to form a registry of practice, and a collection of facts, that must prove of the utmost importance to future inquirers.

---

*Westminster*

*Westminster Hospital, August 29, 1804.*

The established plan of instruction for the Surgeons' pupils of this Hospital will be continued throughout the present season.

Mr. Lynn, Mr. Morel, and Mr. Carlisle, will exhibit and explain the several chirurgical operations; and Mr. Carlisle will deliver occasional lectures on the subjects deemed most useful for hospital students.

Mr. Carlisle will give an introductory lecture at the hospital, on Tuesday, October 2, at twelve o'clock precisely.

For other particulars, apply to Mr. Lynn, Surgeon, Parliament Street; or Mr. Carlisle, Surgeon, Soho Square.

---

*Medical Theatre, St. Bartholomew's Hospital.*

The following Courses of Lectures will be delivered at this Theatre during the ensuing winter.

On the Theory and Practice of Medicine, by Dr. Roberts and Dr. Powell.—Clinical Lectures on select cases occurring in the Hospital, will be occasionally given by Dr. Roberts.

On Anatomy and Physiology, by Mr. Abernethy.

On the Theory and Practice of Surgery, by Mr. Abernethy.

On Chemistry and the Materia Medica, by Dr. Powell.

On Midwifery and the Diseases of Women and Children, by Dr. Thynne.

The Anatomical Lectures will begin on Monday, October 1, at two o'clock, and the other Lectures in the course of the same week.

Further particulars may be known by applying to Mr. Nicholson, at the Apothecary's Shop, St. Bartholomew's Hospital.

---

Mr. Wilson and Mr. Thomas, will commence their Lectures on Anatomy, Physiology, Pathology, and Surgery, at the Theatre, of Anatomy, Great Windmill Street. One Course on October 1, terminating on January 18; and the other Course beginning on January 19, and terminating towards the middle of May. In the October Course they explain the structure of every part of the human body, so as to exhibit a complete view of its Anatomy, as far as it has been hitherto investigated; to which are added, its Physiology and Pathology. In the Spring Course, the structure of the human body is again explained; after which follow, Lectures on the Operations of Surgery, and the Course concludes with the Anatomy of the Gravid Uterus. A Lecture is given daily, from two until four o'clock.

A room is likewise open for Dissections, from nine o'clock in the morning till two in the afternoon, from October 10, till April 20; where regular and full demonstrations of the parts dissected are given; where the different cases in Surgery are explained; the methods of operating shewn on the dead body; and where also the various arts of Injecting and making Preparations are taught.

---

*Theatre*

*Theatre of Anatomy, Blenheim Street, Great Marlborough Street.*

Mr. Brookes will commence his Autumnal Course of Lectures on Anatomy, Physiology, and Surgery, on Monday, October 1, at two o'clock. A suite of commodious apartments, thoroughly ventilated, and replete with every convenience for the purposes of dissecting and injecting, will be open every morning till two o'clock, where Mr. Brookes attends.

Dr. Hooper will commence a Course of Lectures on the Practice of Physic, Materia Medica, and Pharmaceutical Chemistry, on Wednesday, October 3, at Mr. Brookes's Theatre. These Lectures will be delivered every morning, (Sundays excepted) at a quarter before eight o'clock.

Dr. Batty will commence his Autumnal Course of Lectures on the Theory and Practice of Midwifery, and on the Diseases of Women and Children, on Monday, October 8, at half past ten o'clock. These Lectures will be given at Mr. Brookes's Theatre.

Prospectuses and particulars of the different Courses of Lectures delivered at this Medical School, may be had of Mr. Brookes, Blenheim Street; Dr. Hooper, St. Mary-le-bone Infirmary; or of Dr. Batty, Great Marlborough Street.

---

### University of Glasgow.

The Medical Lectures in the University of Glasgow will begin on Tuesday, the 6th of November, at the following hours.

Dietetics, Materia Medica, and Pharmacy, by Dr. Millar, at ten o'clock in the forenoon.

Midwifery, by Mr. Towers, at eleven.

Theory and Practice of Physic, by Dr. Freer, at twelve.

Anatomy and Surgery, by Dr. Jeffrey, at two o'clock in the afternoon.

Chemistry and Chemical Pharmacy, by Dr. Cleghorn, at seven.

Clinical Lectures on the cases of patients in the Royal Infirmary, by Dr. Freer and Dr. Cleghorn. The first Lecture by Dr. Freer, on Tuesday evening, the 13th of November, at six o'clock.

Dr. Brown will commence his Lectures on Botany, about the beginning of May next.

*Regulations enacted by the Senate of the University of Glasgow, respecting Degrees in Medicine.*

1. That before any person can be allowed to be a Candidate for a Degree in Medicine in this University, he shall appear personally before the Senate, and lay before them evidence, that during the space of three years, or sessions of six months each, he has regularly attended in some University or Universities, or in some Medical School or Schools of reputation, the following Medical Classes, viz. Anatomy and Surgery, Chemistry and Pharmacy, the Theory and Practice of Physic, Materia Medica, and Botany.

2. That

2. That he shall bring forward evidence, that, during one year at least, he has attended Medical Classes in this University.

3. That the Candidate shall undergo three separate examinations in private by the Medical Professors of the University; and write a Commentary on an Aphorism of Hippocrates, and another on a case of disease propounded to him by the said Examiners. The first examination shall be on Anatomy and Physiology; the second, on the Theory and Practice of Physic; and the third on Chemistry, Materia Medica, Pharmacy, and Botany.

4. That the Examiners shall report to the Senate their opinion respecting the medical knowledge of the Candidate; and if their report be favourable, his name as a Candidate for a Degree shall be entered in the minutes of Senate, and a day fixed when the Candidate shall read his Commentaries on the Aphorism and Case, and answer such questions on the several branches of medical science, as shall be put to him by the examiners, in presence of the Senate. If the Senate be of opinion that the Candidate has shewn himself worthy of a degree, it shall be conferred in presence of the Senate, by the Vice Chancellor, provided the Candidate has not published a Thesis, which he may or may not do, according to his own option: but if he has published a Thesis, he must defend it, and the degree must be conferred in the Comitia.

5. That the whole of the examinations shall be carried on, and the Commentaries upon the Aphorism and Case must be written in the Latin language.

---

Dr. Bradley will commence his Course of Lectures on the Theory and Practice of Medicine, in the first week of October, at his house, No. 25, Parliament Street.

---

Dr. George Pearson, M. D. F. R. S. senior physician to St. George's Hospital, will commence his Lectures on Physic and Chemistry in the first week of October, as usual.

---

Dr. Badham's Lectures on the Practice of Physic, Chemistry, and Materia Medica, will commence on Wednesday, October 10, at eight o'clock in the morning, at Dr. Crichton's late Laboratory and Lecture Room, Clifford Street, Burlington Gardens, where printed particulars may be had.

---

Dr. Dennison and Dr. Squire will, on Thursday, the 4th of October, begin a Course of Lectures on the Principles and Practice of Midwifery, and the Diseases of Women and Children.

---

Dr. Clarke's Lectures on Midwifery and the Diseases of Women and Children.—The winter courses will begin at Dr. Clarke's House, New Burlington Street, on Thursday, October 4, at a quarter past ten o'clock. Particulars may be known and plans of the Lectures had, by applying to Dr. Clarke, as above.

---

Mr. SYER will commence his Lectures on the Elements and Practice of Midwifery, &c. on Monday, October 1, at six o'clock in the evening; and in future, on Tuesdays, Thursdays, and Saturdays, at the same hour, for the convenience of those gentlemen who attend surgical lectures. The Course will be given at No. 5, Bishopsgate Street, Within.

---

Mr. TAUNTON will commence his Autumnal Course of Lectures on Anatomy, Physiology, and Surgery, at the Finsbury Dispensary, St. John's Square, on Saturday, the 6th of October, 1804. —Practical Anatomy, as usual.

---

Mr. CARPUE will commence his Anatomical Lectures, at his Theatre, Broad Street, Golden Square, on Monday, Oct. 1, 1804.

---

Mr. CHEVALIER, Surgeon to the Westminster General Dispensary, will commence his Autumnal Course of Lectures on the Principles and Operations of Surgery, on Wednesday, October 3, at seven o'clock in the evening, at his house in South Audley Street, Grosvenor Square, where printed particulars may be had.

---

Mr. GILHAM, in a letter to Dr. Bradley, dated Aug. 16, 1804, says, "From my personal knowledge of you, I take the opportunity of noticing the anonymous and improper call on me, in No. 66, of the Medical and Physical Journal, respecting the Death Wound of Sir Ralph Abercrombie; and have only to observe, that if I had considered it of sufficient interest to the public, I should have laid it before them long since."

---

A Correspondent in Bedfordshire has requested us to insert the following Note. "A Subscriber wishes to be informed, through the medium of the Medical Journal, when the posthumous Work of Dr. Garnett will be published?"

---

Mr. ALDER will publish in a few months, in one large volume octavo, Extracts and Observations intended to shew the Nature of the Causes of Epidemic and other Fevers; and also the Meaning which ought to be affixed to the term Putrid Contagion, if we do not entirely abolish it.

Mr. ALDER will also publish, in a few months, in one large volume octavo, a Treatise on Fever, shewing the Genera, Species, and Varieties of it, with the proper Mode of Treatment of each. The above works are intended to begin a Series to complete the design sketched in Mr. A's outlines.

